# Trichrome-positive intrahepatic cytoplasmic globules are potential histopathological clue for COVID-19-induced hepatitis: a case report

**DOI:** 10.1186/s43066-021-00140-5

**Published:** 2021-08-23

**Authors:** Dina Sweed, Mohamed Ramadan El Shanshory, Eman Mohammed Elaskary, Hassnaa Atef Hassan, Enas Sweed, Eman Sweed, Shimaa Abdelsattar, Ahmed Abdelgawad, Asmaa Mosbeh, Heba Abdallah, Shereen El-Mashad, Nermine Ehsan

**Affiliations:** 1 Pathology Department, National Liver Institute, Menoufia University, Shibin el Kom, Egypt; 2 Pediatric Department, Faculty of Medicine, Tanta University, Tanta, Egypt; 3 Radiology Department, Faculty of Medicine, Benha University, Benha, Egypt; 4 Clinical Pharmacology Department, Faculty of Medicine, Menoufia University, Shibin el Kom, Egypt; 5 Clinical Pathology Department, National Liver Institute, Shibin el Kom, Egypt; 6 Clinical Biochemistry, and Molecular Diagnostics Department, National Liver Institute, Menoufia University, Shibin el Kom, Egypt

**Keywords:** Autoimmune hemolytic anemia, Intracytoplasmic globules, Pathology, SARS-CoV-2

## Abstract

**Background:**

Severe acute respiratory syndrome coronavirus 2 (SARS-CoV-2) infection mainly affects respiratory system. Later, liver affection has also been reported in the form of marked elevated liver enzymes. However, the association of coronavirus disease-19 (COVID-19) and autoimmune diseases is not clear.

**Case presentation:**

A female patient with a known history of autoimmune hemolytic anemia (AIHH) for which she was treated with prednisolone was admitted for uncontrolled anemia followed by fever and elevated liver enzymes. All the laboratory and radiological investigations were not typical for COVID-19 or any other etiology. Liver biopsy revealed numerous pale eosinophilic trichrome-positive intracytoplasmic globules. The pathology raised the suspicion for SARS-CoV-2-associated hepatitis, which was confirmed by a positive IgG titer. The patient showed a dramatic improvement on the maintenance dose of prednisolone.

**Conclusions:**

AIHA patients co-infected with SARS-CoV-2 may be at risk of uncontrolled disease and should continue their treatment regimen. Histopathology has a role in the diagnosis of liver affection due to SARS-CoV-2 infection.

## Background

Severe acute respiratory syndrome coronavirus 2 (SARS-CoV-2) is responsible for the coronavirus disease 19 (COVID-19) that started in Wuhan, China, in December 2019 [1]. The virus is transmitted by respiratory droplets and attacks the respiratory system. The typical clinical presentation is high-grade fever and dry cough associated with the characteristic chest imaging. Gastrointestinal and liver symptoms have been reported as the first, or occasionally the only presentation of COVID-19 [2]. Liver affection presents usually in the form of elevated liver enzymes that may progress to fulminant hepatitis [3, 4]. The diagnostic role of liver pathology in the COVID-19 pandemic is very limited. We report a case of SARS-CoV-2 infection in a patient with autoimmune hemolytic anemia (AIHA) presenting with uncontrolled anemia and elevated liver enzymes. The diagnosis was raised based on histopathological findings from liver biopsy.

## Case presentation

**Fig. 1:**
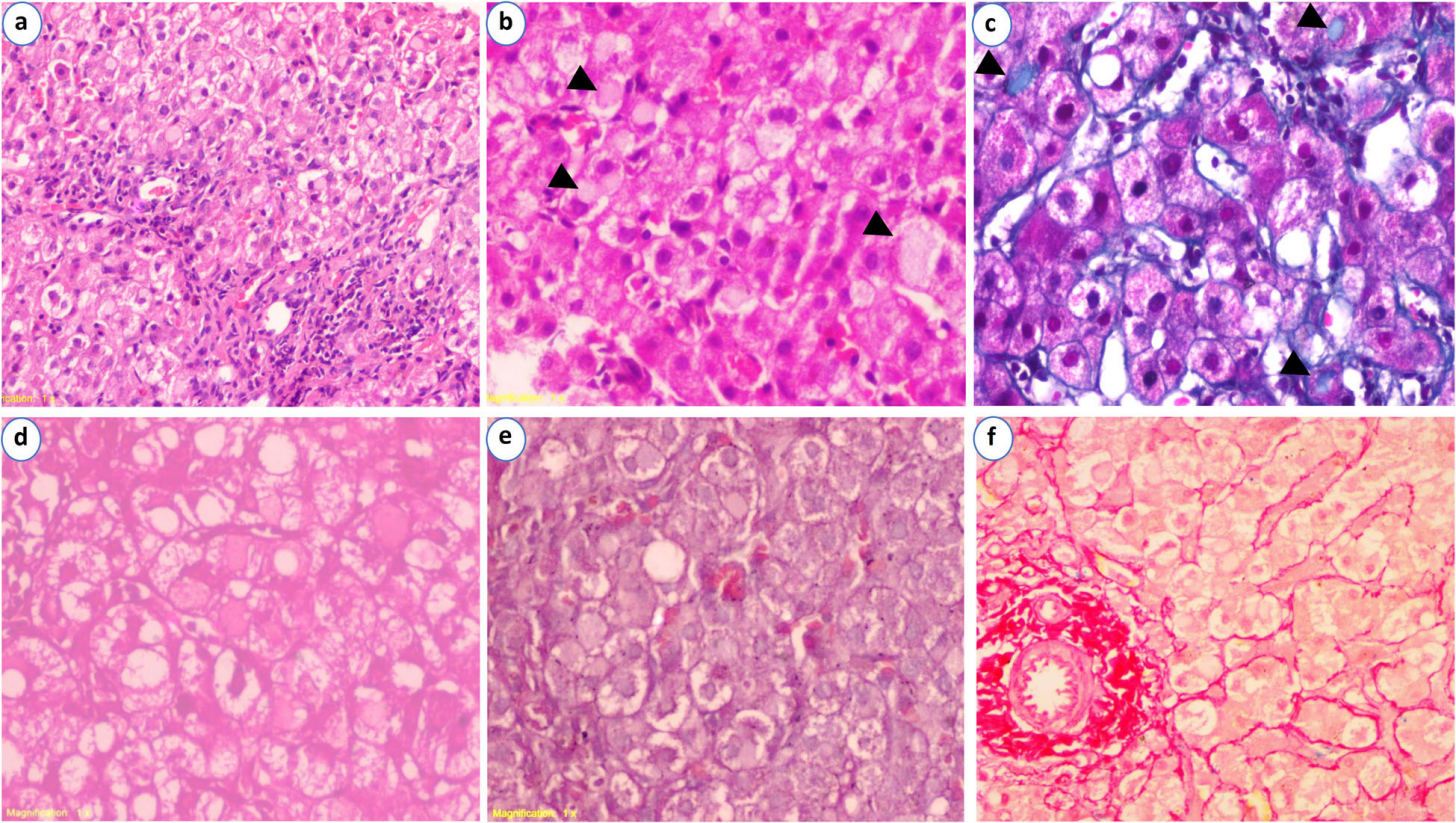
Histopathology of COVID-19-induced hepatitis. **a** Mild expansion of portal tracts by chronic inflammatory cells with mild interface hepatitis and mild periportal fibrosis (H&E × 100). **b** Higher power showing numerous pale intracytoplasmic eosinophilic globules (H&E × 400). **c** The globules were highlighted by trichrome stain as light blue globules (MT × 400). **d** The globules faded after staining with periodic acid-Schiff-diastase stain (PAS-D × 400). **e** Lack of copper associated globules within hepatocytes and negative staining of the globules (Orcein × 400). **f** Lack of iron deposition within hepatocytes and negative staining for the globules (Perls Prussian blue × 400)

A 12-year-old girl was admitted to the hospital for pallor, uncontrolled anemia, and jaundice. The patient had a 5-year history of AIHA, controlled by prednisolone at a daily dose of 20 mg. On day 60 of admission, the patient suffered from fever of 39 ^o^C without any associated respiratory or gastrointestinal symptoms. Her lung examination and chest X-ray were free. Microbiological investigations including Widal test and stool culture were negative for any infection except for salmonella typhi a, and the patient received cephalosporin, with no improvement of fever. Abdominal sonography revealed hepatomegaly and marked splenomegaly. Laboratory investigations were as follows: hemoglobin 7.7 g/dl, platelet 261,000 K/μl, TLC 8400 K/μl, total bilirubin 4.9 mg/dl, direct 1.9 mg/dl, ALT 853 IU/L, AST 692 IU/L, albumin 3.2 g/dl, GGT 103 U/l, ferritin 117 ng/ml, and D-dimer 0.34 ng/ml. Further laboratory tests to declare the etiology were done as follows: autoimmune markers, ANA, ANCA, anti-DNA, and LKM were negative except for ASMA (1/20 titer). In addition, ceruloplasmin and serum copper were normal, and all hepatitis viruses were negative. The history of AIHA raised the possibility of concomitant autoimmune hepatitis or Wilson’s disease. Liver biopsy was indicated as a final approach for diagnosis. The histopathological findings revealed a mild portal tract expansion by mixed inflammatory cells containing few plasma cells, mild interface hepatitis, and mild periportal fibrosis in some portal tracts. There was no thrombosis or sinusoidal fibrin deposition. Hepatic parenchyma showed multiple foci of necroinflammatory activity, ballooning degeneration, and mild macrovesicular steatosis (5%). Numerous pale eosinophilic intracytoplasmic, rounded inclusions of various sizes were seen distributed evenly in the parenchyma. The globules were positive for masson trichrome stain and negative for periodic acid Schiff–diastase (PAS-D), orcein, and perls stains (Fig. 1). Based on a previous case report that highlighted those globules as a characteristic finding for SARS-CoV-2 infection [5], the pathological diagnosis raised the suspicion for post-COVID-19 hepatitis. Therefore, serum IgG and IgM were done and revealed a positive IgG result for SARS-CoV-2 infection (14.5, positive if > 1). Chest CT was not advisable due to a time lag between the onset of symptoms and the clinical suspicion with the rapid resolution of patches in the pediatric age. Two weeks later, there was a dramatic improvement of liver enzymes, ALT 279 IU/L, AST 231 IU/L, albumin 4.3 g/dl, GGT 53 U/l, ALP 140 U/l, hemoglobin 8.7 g/dl, and TLC 8200 K/μl, and serum fibrinogen 298 mg/dL. After another 2 weeks, ALT was 107 IU/L and AST was 104 IU/L. The patient was followed up for 6 months and maintained her daily dose of corticosteroid with no further intervention.

## Discussion

Liver affection associated with SARS-CoV-2 has been reported either as unexplained elevated liver enzymes or as postmortem findings [6]. However, few cases of SARS-COV-2 infection associated fulminant hepatitis have been reported [3, 4]. The COVID-19 associated liver injury is multifactorial resulting from the cross talk of multi-organs injury, direct viral cytopathic effect, inflammatory and cytokine storm, thrombo-ischemic factors, drug-induced, and background liver disease [7]. The viral direct cytopathic effect could be related to the wide distribution of SARS-CoV-2 host receptors in liver tissue, specifically angiotensin-converting enzyme 2 (ACE2) [8]. However, the mechanism is not fully understood. In this case, the patient was admitted for uncontrolled AIHA for which she was given prednisolone. Two months later, the patient developed persistent fever not responding to treatment followed by elevated liver enzymes. The patient’s laboratory and radiological findings were not typical for COVID-19, autoimmune hepatitis, or Wilson’s disease. Liver biopsy was the final step in the diagnosis and management. The pathological findings showed characteristic pale intracytoplasmic globules of ground-glass appearance. The globules were positive for masson trichrome stain (fibrin stain) and negative for PAS-D, orcein, and perls stains. The differential diagnosis of ground-glass hepatocytes includes HBsAg in HBV infection, alpha-1-antripysin deficiency, Lafora’s disease, type IV glycogenosis, and cyanamide alcohol aversion therapy. They have also been described in patients under polypharmacotherapy [9]. All these possibilities were ruled out based on clinical, laboratory data, and histopathological interpretations. Interestingly, Fraga et al. reported deposition of type II fibrinogen in a COVID-19 patient who presented with severe hepatitis [5]. The mechanism of elevated fibrinogen level is related to the release of the acute-phase inflammatory mediators, e.g., interleukin 6 (IL-6) [10]. The normal serum fibrinogen, in this case, ruled out the possibility of genetic fibrinogen storage disease, which is usually associated with hypofibrinogenemia [5].

A spectrum of SARS-CoV-2 infection complications includes multiple autoimmune disorders, AIHA mainly in high-risk patients [11]. However, a case of SARS-CoV-2-infected patient developed AIHA in the absence of any underlying predisposition [12]. The possible mechanism is the cytokine storms with release of interleukin 6 [13]. In this case, the patient’s hemoglobin and liver function were dramatically improved on the maintenance dose of prednisolone. A recent clinical trial postulated the efficacy of corticosteroids in managing COVID-19 patients; however, its impact on viral clearance and risk of other infections is a matter of controversy [14–16].

## Conclusions

AIHA patients co-infected with SARS-CoV-2 might be at increased risk of uncontrolled disease and should maintain their treatment regimen; however, it does not associate with poor outcomes. Corticosteroid may play a role in preventing disease progression in this case. The pathological findings of trichrome positive intracytoplasmic globules could be suggestive for SARS-CoV-2 induced hepatitis, especially in the setting of an ongoing pandemic and could be a diagnostic clue for liver affection.

## Data Availability

Availability of data and materials are available from the corresponding author on reasonable request.

## Abbreviations

SARS-CoV-2: Severe acute respiratory syndrome coronavirus 2
AIHH: Autoimmune hemolytic anemia
COVID-19: Coronavirus disease 19
PAS-D: Periodic acid Schiff–diastase
ACE2: Angiotensin-converting enzyme 2
IL-6: Interleukin 6
